# Child and maternal benefits and risks of caseload midwifery – a systematic review and meta-analysis

**DOI:** 10.1186/s12884-023-05967-x

**Published:** 2023-09-15

**Authors:** Lotta Wassén, Beata Borgström Bolmsjö, Sophia Frantz, Anna Hagman, Marie Lindroth, Christine Rubertsson, Annika Strandell, Therese Svanberg, Anna Wessberg, Susanna M. Wallerstedt

**Affiliations:** 1 Department of Obstetrics and Gynecology, Sahlgrenska University Hospital, Gothenburg, Sweden; 2 Department Research and education, HTA syd, Skåne University Hospital, Lund, Sweden; 3 Department of Clinical Sciences Malmö, Lund University, Malmö, Sweden; 4 Clinical Physiology and Nuclear Medicine Unit, Department of Translational Medicine, Lund University, Malmö, Malmö, Sweden; 5 Regional Healthcare, Region Västra Götaland, Gothenburg, Sweden; 6 Midwifery Clinic in Primary Care, Region Skåne, Malmö, Sweden; 7 Department of Health Science, Medical faculty, Lund University, Lund, Sweden; 8 HTA-centrum, Sahlgrenska University Hospital, Region Västra Götaland, Gothenburg, Sweden; 9 Medical library, Sahlgrenska University Hospital, Region Västra Götaland, Gothenburg, Sweden; 10 Department of Pharmacology, Sahlgrenska Academy, University of Gothenburg, Box 431, Gothenburg, SE-405 30 Sweden

**Keywords:** Caseload midwifery, GRADE, Meta-analysis, Care model, Systematic review

## Abstract

**Background:**

It has been reported that caseload midwifery, which implies continuity of midwifery care during pregnancy, childbirth, and the postnatal period, improves the outcomes for the mother and child. The aim of this study was to review benefits and risks of caseload midwifery, compared with standard care comparable to the Swedish setting where the same midwife usually provides antenatal care and the checkup postnatally, but does not assist during birth and the first week postpartum.

**Methods:**

Medline, Embase, Cinahl, and the Cochrane Library were searched (Nov 4th, 2021) for randomized controlled trials (RCTs). Retrieved articles were assessed and pooled risk ratios calculated when possible, using random-effects meta-analyses. Certainty of evidence was assessed according to GRADE.

**Results:**

In all, 7,594 patients in eight RCTs were included, whereof five RCTs without major risk of bias, including 5,583 patients, formed the basis for the conclusions. There was moderate certainty of evidence for little or no difference regarding the risk of Apgar ≤ 7 at 5 min, instrumental birth, and preterm birth. There was low certainty of evidence for little or no difference regarding the risk of perinatal mortality, neonatal intensive care, perineal tear, bleeding, and acute caesarean section. Caseload midwifery may reduce the overall risk of caesarean section. Regarding breastfeeding after hospital discharge, maternal mortality, maternal morbidity, health-related quality of life, postpartum depression, health care experience/satisfaction and confidence, available studies did not allow conclusions (very low certainty of evidence). For severe child morbidity and Apgar ≤ 4 at 5 min, there was no literature available.

**Conclusions:**

When caseload midwifery was compared with models of care that resembles the Swedish one, little or no difference was found for several critical and important child and maternal outcomes with low-moderate certainty of evidence, but the risk of caesarean section may be reduced. For several outcomes, including critical and important ones, studies were lacking, or the certainty of evidence was very low. RCTs in relevant settings are therefore required.

**Supplementary Information:**

The online version contains supplementary material available at 10.1186/s12884-023-05967-x.

## Introduction

Midwife-led continuity models, compared with other models of care for childbearing women, are reportedly favorable regarding several mother and child outcomes, including a reduced risk of instrumental vaginal birth, a reduced risk of preterm birth (< 37 weeks), and a reduced risk of fetal loss before and after 24 weeks including neonatal death [1]. Midwife-led continuity models include caseload midwifery, a model in which a primary midwife, within a team of midwives provides care to a load of cases during antenatal, intrapartum, and postpartum care. Caseload midwifery has been established in several countries, including Denmark [2], the Netherlands [3], England [4], and Australia. [5].

In Sweden, about 115,000 children are born each year. In standard care, midwives provide antenatal, intrapartum and postpartum care. When complications arise, other professionals, e.g. physicians, are consulted. When possible, the same midwife provides care during pregnancy and postnatal follow-up after the first week, whereas hospital-employed midwives provide care during childbirth and the first week postpartum. Almost all childbirths in Sweden take place in hospitals, and midwives assist the women during vaginal birth. According to the Swedish Pregnancy Register [6], the prevalence of caesarean section was 18.5% in 2021, and perinatal death 0.35%.

As there is a shortage of midwives in Swedish labor wards, caseload midwifery has gained increased interest as an alternative model of care. Indeed, an integrative literature review revealed several factors contributing to job satisfaction for midwives in caseload models of care, including the ability to build relationships with women, the flexibility and control, as well as the professional autonomy and identity [7]. The midwife-woman relationship has also been explored to be of value for the woman, including themes of personalized care, trust, and empowerment [8]. As far as we are aware, however, the scientific literature regarding effects for mother and child in the caseload model of care, compared with models comparable to Swedish standard care, has not previously been reviewed. Therefore, this systematic review was performed to evaluate benefits and risks, for the mother and the child, of caseload midwifery compared with other models of care where the same midwife generally provides ante- and postnatal care but does not assist during childbirth and the first week postpartum.

## Methods

This systematic review was performed according to the established routines at the regional health technology assessment (HTA) center (HTA-centrum) in Region Västra Götaland, Sweden, and reported according to the PRISMA guidelines [9]. The aim was defined in a PICO (Participants, Intervention, Comparison, Outcome). Participants (P) were pregnant women, without planned home birth, and their child/ren. The intervention (I) was caseload midwifery. For scheduling reasons, we considered it reasonable that caseload midwifery could include quite large caseload teams. Therefore, we did not exclude studies based on the size of the team. The comparison (C) was standard care similar to the Swedish model, i.e. with one maternal care midwife during antenatal and postnatal care, and hospital care midwives during birth and the week afterwards. As we did not expect to identify studies exactly matching the Swedish model, we decided to include studies in which midwives performed a considerable part of the antenatal care. When not clearly reported, we decided to include rather than to exclude, and to handle this uncertainty in the directness assessments. Outcomes (O) included child outcomes: perinatal mortality, severe morbidity (e.g. brain injuries, body injuries, or severe infection), Apgar ≤ 4 at 5 min, Apgar ≤ 7 at 5 min, neonatal intensive care, and breastfeeding after discharge, as well as maternal outcomes: mortality, intensive care, health-related quality of life (HRQL), perineal tear (grade I-IV and III-IV), bleeding, caesarean section (total and acute), instrumental birth, postpartum depression, preterm birth, and health care experience/satisfaction/confidence. Perinatal mortality and morbidity, as well as maternal mortality and intensive care were considered critical for decision-making. At the other end, breastfeeding and health care experience/satisfaction/confidence were considered useful, and the remaining outcomes important for decision-making. Regarding preterm birth, this outcome may not be directly affected by adding continuity of carer during intrapartum care when ante- and postnatal care is already performed by the same midwife. However, as this outcome was included in a previous systematic review within the field [1], and as caseload models may have indirect effects, it was considered an important outcome. Publications were restricted to randomized controlled trials (RCTs) and languages to English, Swedish, Danish, and Norwegian. The preparatory work for this review was performed within an HTA [10].

### Literature search and study selection

On November 4th, 2021, two medical librarians performed systematic searches in Medline, Embase, Cinahl, and the Cochrane Library. Reference lists of relevant articles were scrutinized for additional references. To identify ongoing studies, we performed a search in Clinicaltrials.gov and WHO International Clinical Trials Registry Platform (ICTRP, March 28th, 2022). Search strategies are provided in Additional file 1.

Two authors screened identified abstracts and those that clearly did not meet the PICO criteria were excluded in a consensus discussion. When there were uncertainties regarding inclusion/exclusion, the full text was retrieved and assessed independently by at least two authors. Inclusion/exclusion according to the PICO was then decided in a consensus meeting. As rostered midwives may provide ante- and postnatal care in Sweden, although continuity of carer is intended, we included studies where the circumstances in the control group in consensus discussions were considered sufficiently similar to the Swedish model, and, as described previously, handled potential uncertainties in the assessments of directness. For articles excluded in consensus, after full-text reading, reasons for exclusion were recorded. The remaining studies were included in the systematic review.

### Data extraction and study assessments

Data were extracted from the studies by two authors independently and were subsequently checked by the other authors. Data extraction included the participants studied, the number and characteristics of individuals in the intervention and control groups, and the results regarding the outcomes selected in the PICO.

Each study was critically and independently appraised by at least two authors, focusing on the domains directness and risk of bias according to the checklist for assessing RCTs used by HTA-centrum [11]. Regarding directness, we assessed to what extent the studied population, intervention, comparison, and outcome measures corresponded to the question at issue. Regarding risk of bias, we focused on selection bias (random sequence generation and concealed allocation, respectively), performance bias, detection bias, attrition bias, reporting bias, and other bias. Subsequently, all authors discussed the assessments and categorized, in consensus, each study overall as having no or minor problems (+), some problems (?), or major problems (-) in the domains directness and risk of bias. The certainty of evidence was assessed using the Grading of Recommendations Assessment, Development and Evaluation (GRADE) in which potential issues regarding risk of bias (study limitations), consistency, directness, and precision are considered across studies, to appraise the overall quality of evidence [12].

### Statistics

When three or more RCTs provided data regarding a specific outcome, we performed random-effects meta-analyses using the software Review Manager (RevMan) version 5.4.1 (The Nordic Cochrane Centre, The Cochrane Collaboration, Copenhagen, Denmark) to obtain risk ratios with 95% confidence intervals (CIs). When merely two RCTs provided data regarding a specific outcome, we assessed if these were sufficiently clinically homogenous to allow pooling. We consistently used the number of randomized individuals as denominator. When the pooled result was statistically significant, i.e., the 95% CI of the risk ratio did not cover 1, we calculated the risk difference with 95% CI to gain knowledge about the magnitude of the effect. According to the predefined analysis plan, RCTs without major risk of bias were compiled and formed the basis for the conclusions. Beforehand, we also planned subgroup meta-analyses including pregnant women with fear of childbirth; crude analyses in a small non-randomized study suggest that women with fear may benefit from a continuity model of care [13]. When relevant, we also performed sensitivity meta-analyses to investigate the robustness of the results. Heterogeneity was assessed with I^2^.

## Results

**Fig. 1:**
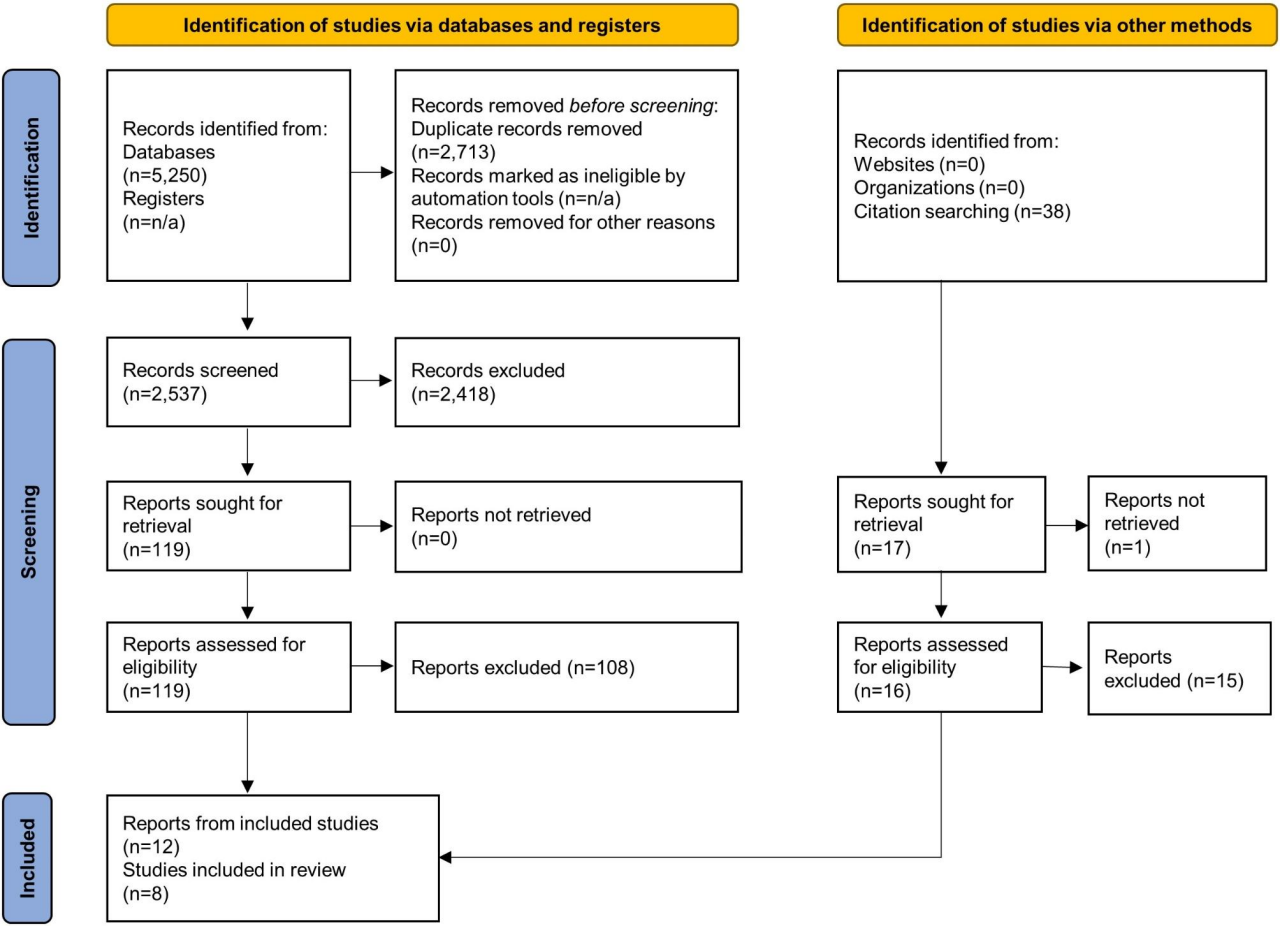
PRISMA flowchart

After removal of duplicates, the literature search identified 2,575 unique publications, and 12 publications, based on eight RCTs, were included in this systematic review (Fig. 1) [14–25]. Publications excluded after full-text reading by the authors, as well as the reasons for excluding them, are presented in Supplemental Table 1.

### Study characteristics

In all, seven RCTs with individual randomization [14, 17, 19–21, 23, 25] and one RCT with cluster-randomization [24] fulfilled the PICO of this review, all non-blinded and including a total of 7,594 women, (Table 1). Four RCTs were performed in Australia [17, 19, 21, 25], three in England [14, 20, 24], and one in New Zealand [23]. Three RCTs were assessed to have major risk of bias [19, 23, 24], and the remaining five, with associated publications, formed the basis for the conclusions [14, 17, 20, 21, 25]. Reasons underlying the directness and risk of bias assessments are described in Supplemental Table 2.

**Table 1:** Characteristics of included RCTs

First author Year	Country (Acronym)	Participants		Caseload midwifery	Standard care
		Characteristics	I vs. C n age		
Fernandez Turienzo 2020 [14]	England (POPPIE)	Women at increased risk of preterm birth	169 vs. 165 32 vs. 32 yrs.	One team Six midwives 35 women/midwife/year	Rostering midwife, no planned continuity
Fernandez Turienzo 2021 [15]					
Forster 2016 [16]	See McLachlan 2012				
Homer 2001 [17]	Australia (STOMP)	Women without significant disease	550 vs. 539 28 vs. 28 yrs.	One team Six midwives 50 women/midwife/year	Hospital-based antenatal care, midwives and/or obstetricians/GPs
Homer 2002 [18]					
Homer 2021 [19]	Australia	Women with one previous CS, low-risk pregnancy	108 vs. 110 31 vs. 31 yrs.	NR	Midwife team antenatally, rostering midwives at birth
Marks 2003 [20]	England	Women with ≥ 1 episode of major depressive disorder	44 vs. 43 32 vs. 32 yrs.	One team Six midwives Number of women/midwife/year NR	Midwives or GPs
McLachlan 2012 [21]	Australia (COSMOS)	Women with low-risk pregnancy	1156 vs. 1158 32 vs. 32 yrs.	One team 12 midwives 45 women/midwife/year	Antenatally: midwives (78%), GPs (15%), obstetric trainee (2%), other (5%)
McLachlan 2016 [22]					
Morrison 2002 [23]	New Zealand	Women with diabetes	140 vs. 144 33 vs. 32 yrs.	One team Three midwives Number of women/midwife/year NR	Antenatally: One of two dedicated diabetic clinic midwives
North Staffordshire 2000 [24]	England	No exclusions^1^	770 vs. 735 28 vs. 28 yrs.	Three caseload areas, 26 midwives Two-three midwives/team 35–40 women/midwife/year	Shared care: GP/midwife
Tracy 2013 [25]	Australia (M@NGO)	Women of any risk, without a planned elective CS	871 vs. 877 32 vs. 32 yrs.	Number of teams NR Four midwives/team 40 women/midwife/year	Shared care: GP/midwife

^1^cluster-randomized

C = comparison (standard care), COSMOS = COmparing Standard Maternity care with One-to-one midwifery Support, CS = caesarean section, I = intervention (caseload midwifery), GP = general practitioner, M@NGO = Midwives @ New Group practice Options, NR = not reported, POPPIE = Pilot study Of midwifery Practice in Preterm birth Including women’s Experiences, RCT = randomized controlled trial, STOMP = St George Outreach Maternity Project

### Child outcomes

**Fig. 2:**
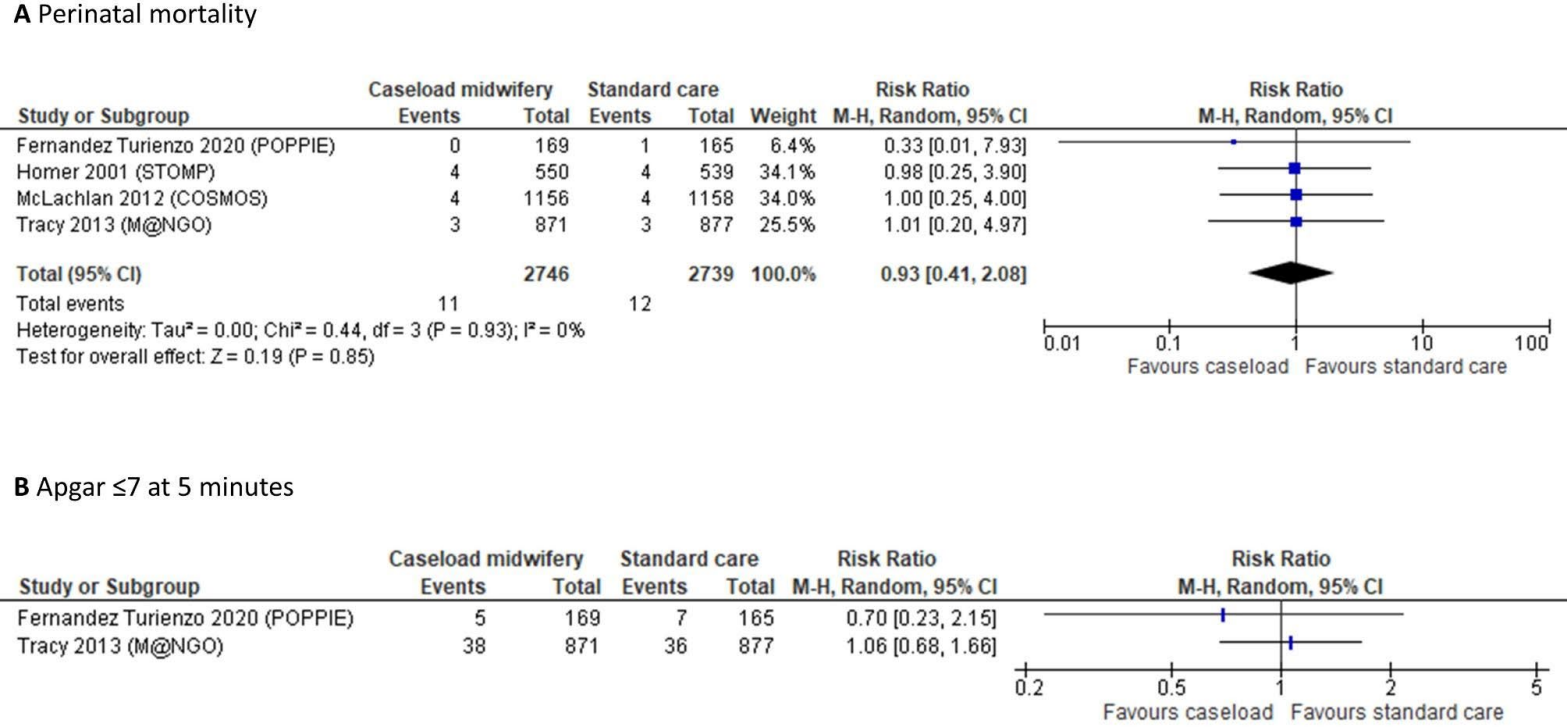
Forest plots of RCTs reporting child outcomes, summarized in meta-analyses if not deemed clinically heterogeneous. **A**: perinatal mortality (absolute risk in standard care ranged from 0.3–0.7%). **A**: Apgar ≤ 7 at 5 min (absolute risk in standard care 4% in both studies)

Results for each RCT presenting data for the studied outcomes are presented in Table 2, and forest plots in Fig. 2. Regarding perinatal mortality, four RCTs without major risk of bias, including 5,465 women, were pooled resulting in a risk ratio of 0.93 (95% CI: 0.41 to 2.08). No data were available regarding severe morbidity and Apgar ≤ 4 at 5 min. Regarding Apgar ≤ 7 at 5 min, two RCTs without major risk of bias contributed data. They were not pooled as they were considered clinically heterogeneous; one study included women at increased risk of preterm birth [14], and the other women of any risk without planned caesarean section [25]. As one additional study reported Apgar < 7 instead of ≤ 7 at 5 min [21], we performed a sensitivity meta-analysis of the three trials, resulting in a risk ratio of 0.92 (95% CI: 0.62 to 1.30; I^2^: 0%). Regarding neonatal intensive care, two RCTs provided unpoolable data [14, 21], none reporting statistically significant differences and none separating routine surveillance from care due to child health issues.

**Table 2:** Results and assessments of included RCTs

First author Year	Child outcomes I vs. C n/n with available data (%)	Maternal outcomes I vs. C n/n with available data (%)	Overall assessment^1^	
			Directness	Risk of bias
Fernandez Turienzo 2020 [14]	• Perinatal mortality: 0/168 (0) vs. 1/163 (0.6) • Apgar ≤ 7 at 5 min: 5/160 (3.1) vs. 7/159 (4.4) • NICU, mean days ± SD: 7.1 ± 14.7 vs. 1.1 ± 3.4 • Breastfeeding, initiation: 133/161 (81) vs. 118/158 (75); at discharge: 112/161 (70) vs. 89/158 (57)	• Mortality: 0/168 vs. 1/163 • Intensive care: 1/168 (0.6) vs. 0/163 (0) • Perineal tear, I-IV: 36/162 (22) vs. 45/160 (28); III-IV: 2/162 (1.2) vs. 3/160 (1.9) • Bleeding, > 1000 ml: 2/168 (1.2) vs. 1/163 (0.6) • CS, total: 51/162 (31) vs. 49/160 (31); acute NR • Instrumental birth: 14/162 (9) vs. 12/160 (8) • Preterm birth: 31/168 (18) vs. 19/163 (12) • Feasibility, assisted at delivery by primary caseload midwife: 95/168 (57); a midwife in the caseload team: 136/168 (81)	?	?
Fernandez Turienzo 2021 [15]	-	• HRQL, physical health^2^: 50.43 vs. 15.80, P = 0.36; mental health^2^: 14.65 vs. 14.76, P = 0.85 • Health care experience/satisfaction, LAS^3^, mean ± SD: 52.12 ± 13.09 vs. 50.69 ± 13.13, NS • Health care confidence, TNS^4^, mean ± SD: 28.89 ± 2.01 vs. 24.68 ± 5.68, MD (95% CI): -4.21 (-5.44; -2.97)	?	-
Forster 2016 [16]		• Feasibility, assisted at delivery by primary caseload midwife: 573/981 (58); a midwife in the caseload team 889/981 (91) • Health care experience/satisfaction, proportional OR (95% CI)^5,^ pregnancy: 3.35 (2.79; 4.03); labor/birth: 2.13 (1.78; 2.56); postnatally: 3.19 (2.64; 3.85)	?	-
Homer 2001 [17]	• Perinatal mortality: 4/550 (0.7) vs. 4/539 (0.7)	• Bleeding, non-specified postpartum hemorrhage: 31/550 (6) vs. 26/539 (5) • CS, total: 73/550 (13) vs. 96/539 (18); acute: 52/550 (9) vs. 62/539 (12) • Instrumental birth: 71/550 (13) vs. 63/539 (12)	?/-	?
Homer 2002 [18]	-	• Feasibility, assisted at delivery by a midwife in the caseload team: 435/550 (79)	?	?
Homer 2021 [19]	• Perinatal mortality: 0/108 (0) vs. 0/110 (0) • Breastfeeding at discharge: 123/134 (88) vs. 122/138 (88)	• Mortality: 0/108 vs. 0/110 • CS, total: 78/108 (72) vs. 74/110 (67), acute NR • Instrumental birth: 12/108 (11) vs. 16/110 (15)	?/-	-
Marks 2003 [20]	-	• Postpartum depression: 10/44 (23) vs. 10/43 (23) (any psychiatric illness)	-	?
McLachlan 2012 [21]	• Perinatal mortality: 4/1146 (0.3) vs. 4/1151 (0.3) • Apgar < 7 at 5 min: 15/1112 (1.4) vs. 20/1080 (1.9) • NICU: 15/1139 (1.3) vs. 20/1137 (1.9)	• Mortality: 0/1142 vs. 0/1144 • Perineal tear, I-IV: 343/696 (49) vs. 301/679 (44); III-IV: 26/693 (4) vs. 20/679 (3) • Bleeding, > 1000 ml: 53/1142 (5) vs. 65/1144 (6) • CS, total: 221/1142 (19) vs. 285/1144 (25); acute: 186/1142 (16) vs. 245/1144 (21) • Instrumental birth: 202/1142 (18) vs. 222/1144 (19) • Preterm birth: 42/1111 (4) vs. 45/1086 (4) • Feasibility, assisted at delivery by primary caseload midwife: 650/1142 (57); a midwife in the caseload team 1016/1142 (89)	?	+
McLachlan 2016 [22]	-	• Health care experience/satisfaction^6^: 697/979 (71) vs. 516/824 (63)	?	-
Morrison 2002 [23]	• Perinatal mortality: 2/134 (1.5) vs. 0/138 (0) • Breastfeeding at discharge: 123/134 (92) vs. 122/138 (88)	• CS, total: 47/134 (35) vs. 49/138 (35); acute: 40/134 (30) vs. 38/138 (28) • Instrumental birth: 20/134 (15) vs. 15/138 (11) • Health care experience/satisfaction^7^: 111/126 (88) vs. 103/127 (81), NS	-	-
North Staffordshire 2000 [24]	• Perinatal mortality: 6/770 (0.7) vs. 11/735 (1.5)	• Mortality: 0/770 vs. 0/735 • Perineal tear, I-IV: 248/770 (32) vs. 221/735 (30); III-IV NR • CS, total: 137/770 (18) vs. 128/735 (17); acute: 62/770 (8) vs. 76/735 (10) • Instrumental birth: 74/770 (10) vs. 84/735 (11) • Feasibility, assisted at delivery by a midwife in the caseload team: 696/770 (95)	-	-
Tracy 2013 [25]	• Perinatal mortality: 3/871 (0.3) vs. 3/877 (0.3) • Apgar ≤ 7 at 5 min: 38/871 (4) vs. 36/877 (4) • Breastfeeding at discharge: 776/871 (89) vs. 747/877 (85); after 6 weeks: 509/567 (90) vs. 388/440 (88); after 6 months: 396/546 (73) vs. 279/398 (70)	• Mortality: 0/871 vs. 0/877 • Perineal tear, I-IV: 343/696 (49) vs. 301/679 (44); III-IV: 26/693 (4) vs. 20/679 (3) • Bleeding, > 1000 ml: 28/820 (3) vs. 43/791 (5) • CS, total: 183/871 (21) vs. 204/877 (23); acute: 114/871 (13) vs. • 110/877 (13) • Instrumental birth: 172/871 (20) vs. 171/877 (19) • Preterm birth: 39/871 (4) vs. 51/877 (6)	?	?

^1^ + = no or minor problems; ?=some problems; – = major problems; for directness and risk of bias issues, see Table S2

^2^PROMIS-10 instrument: mean: 50 for a United States general population, higher scores indicate better outcome

^3^LAS instrument: ten questions, experience of control during delivery, from 1: never or almost never, to 7: almost all of the time

^4^TNS instrument, adapted for midwives: five questions on confidence in midwife over pregnancy and delivery, from 1 = never, to 5 = always

^5^Response to one question “Overall, how would you describe your care in…”, from 1 = very poor, to 7 = very good

^6^Response to one question “Overall experience of childbirth”, from 1 = very negative, to 7 = very positive, proportion responding 6 or 7 presented

^7^Proportion “very satisfied with care”

C = comparison (standard care), CI = confidence interval, CS = caesarean section, HRQL = health-related quality of life, I = intervention (caseload midwifery), LAS = Labour Agentry Scale, MD = mean difference, NICU = neonatal intensive care unit, NR = not reported, NS = non-significant, OR = odds ratio, PROMIS = Patient reported outcomes measurement information system, RCT = randomized controlled trial, SD = standard deviation, TNS = Trust in Nurses Scale

One RCT reported breastfeeding six weeks and six months after discharge [25], with no significant differences between the groups. There were many dropouts in the intervention and comparison groups, 35% versus 50% at six weeks, and 37% versus 55% at six months respectively. Pooling results from two RCTs without major risk of bias regarding breastfeeding at discharge [14, 25] resulted in a risk ratio of 1.11 (95% CI: 0.94 to 1.31; I^2^: 71%).

Reasons for downgrading in the GRADE process are described in Supplemental Table 3. Comparing caseload midwifery with standard care, there may be little or no difference in perinatal mortality (⊕⊕ΟΟ). Concerning child morbidity, no data were available regarding the risk of severe morbidity and Apgar ≤ 4 at 5 min. Further, there was probably little or no difference regarding the risk of Apgar ≤ 7 at 5 min (⊕⊕⊕Ο) and there may be little or no difference in the risk of admission to neonatal intensive care (⊕⊕ΟΟ). Regarding breastfeeding after discharge, the certainty of evidence was assessed as very low, not allowing conclusions (⊕ΟΟΟ).

### Maternal outcomes

**Fig. 3:**
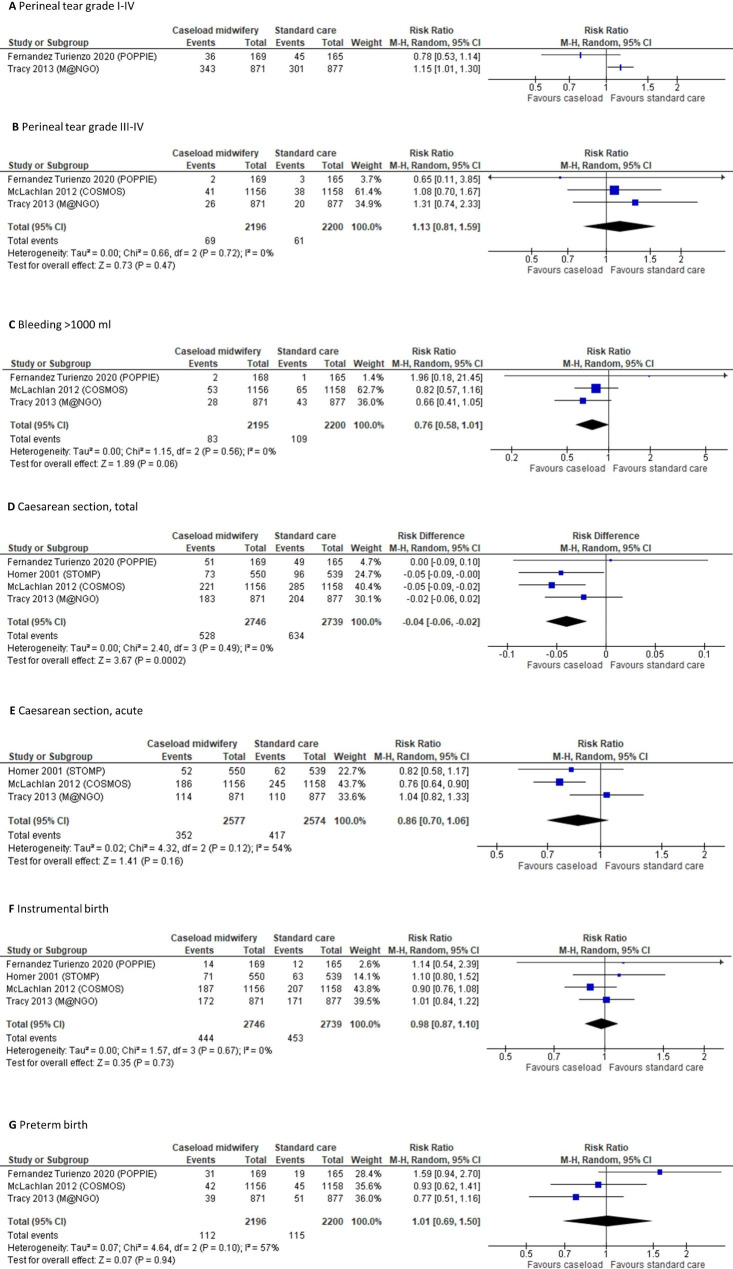
Forest plots of RCTs reporting maternal outcomes, summarized in meta-analyses if not deemed clinically heterogeneous. **A**; perineal tear grade I-IV (absolute risk in standard care: 27% and 34%). **B**: perineal tear grade III-IV (absolute risk in standard care ranged from 1.8–3.3%). **C**: bleeding > 1000 ml (absolute risk in standard care ranged from 0.6–5.6%). **D**: total caesarean section (absolute risk in standard care ranged from 18–31%). **E**: acute caesarean section (absolute risk in standard care ranged from 12–21%). **F**: instrumental birth (absolute risk in standard care ranged from 7–19%). **G**: preterm birth (absolute risk in standard care ranged from 4–12%)

Results for each study presenting data for the studied maternal outcomes are presented in Table 2, and forest plots in Fig. 3. Regarding mortality, five RCTs reported that no deaths occurred [14, 19, 21, 24, 25]. Regarding intensive care, one event, due to sickle cell crisis, was reported in one RCT [14]. HRQL was reported in one RCT, with no significant differences between the comparison groups [14].

Regarding perineal tears grade I-IV, two RCTs without major risk of bias reported results. They were not pooled as they were considered clinically heterogeneous. In one RCT [25], but not the other [14], a statistically significant difference favoring standard care was found. Regarding grade III-IV perineal tears, three RCTs without major risk of bias were pooled resulting in a risk ratio of 1.13 (95% CI: 0.81 to 1.59). In a sensitivity meta-analysis restricted to women with vaginal birth, the risk ratio was 1.08 (95% CI: 0.77 to 1.51; I^2^: 0%). Episiotomy was reported in four RCTs, with frequencies in standard care varying between 9% and 29% [14, 21, 24, 25]. Regarding bleeding > 1000 ml, three RCTs without major risk of bias [14, 21, 25] could be pooled resulting in a risk ratio of 0.76 (95% CI: 0.58 to 1.01). No RCT reported bleedings requiring transfusion.

Caesarean section was reported in seven RCTs, four of which without major risk of bias [14, 17, 21, 25]. Pooling caesarean section overall, the risk ratio was 0.84 (95% CI: 0.75 to 0.94), with absolute risks in standard care varying between 18% and 31% and a pooled risk difference of -4.0 (95% CI: -6.1 to -1.9) percentage units. Three RCTs without major risk of bias reported acute caesarean sections [17, 21, 25], with a pooled risk ratio of 0.86 (95% CI: 0.70 to 1.06).

Instrumental birth was reported in seven RCTs, four of which without major risk of bias [14, 17, 21, 25]. Pooling these RCTs resulted in a risk ratio of 0.98 (95% CI: 0.87 to 1.10). Postpartum depression was reported in one small RCT, with similar event rates in the comparison groups [20]. Preterm birth was reported in four RCTs, three of which without major risk of bias [14, 21, 25]. Pooling these RCTs resulted in a risk ratio of 1.01 (95% CI: 0.69 to 1.50).

Parent experience/satisfaction was reported in four publications [15, 16, 22, 23], based on three RCTs [14, 21, 23]. All four publications were assessed to have major risk of bias. Experience/satisfaction did not differ between the groups in two RCTs [14, 23], but was reported to favor caseload midwifery in one [16]. Confidence, measured as trust in midwife, was reported in one RCT with major risk of bias, favouring the caseload model of care [15].

Reasons for downgrading the certainty of evidence in the GRADE process are described in Supplemental Table 3. The certainty of evidence regarding maternal mortality maternal intensive care, HRQL, and postpartum depression was assessed as very low, not allowing conclusions (⊕ΟΟΟ). There may be little or no difference regarding perineal tears (I-IV and III-IV, respectively) and bleedings (all ⊕⊕ΟΟ). Caseload midwifery may decrease the risk of caesarean section overall, but there may be little or no difference regarding acute caesarean section (both ⊕⊕ΟΟ). The risk of instrumental birth and preterm delivery is probably not affected (both ⊕⊕⊕Ο).

Regarding feasibility, RCTs without major risk of bias reported that 56% [14] or 57% [21] of the women were assisted by their primary caseload midwife during birth, and 79% [18], 81% [14], and 89% [21], respectively, by a midwife in the caseload team. No studies reported results separately for women with fear of birth.

### Ongoing studies

Out of 115 trials identified in Clinical Trials.gov and 198 in ICTRP, no one fulfilled our PICO.

## Discussion

This quantitative evidence synthesis shows, in general, little or no difference between caseload midwifery and standard care comparable to the Swedish setting, where the same midwife generally provides antenatal care and postnatal checkup, but other midwives assist at birth and the first week postpartum. For child outcomes, there may be little or no difference regarding perinatal mortality and neonatal morbidity. No RCTs reported severe neonatal morbidity, and available evidence did not allow conclusions regarding breastfeeding after hospital discharge. Regarding maternal outcomes, the present review shows that the risk of preterm birth, as well as instrumental birth, is probably not affected. There may be little or no difference in the risk of perineal tear and bleeding. Caseload midwifery may, however, reduce the incidence of caesarean section. No conclusions could be drawn regarding maternal mortality, intensive care, HRQL, postpartum depression, and health care experience/satisfaction/confidence.

Our results differ in several ways from the Cochrane review that was also based on RCTs [1]. In contrast to their findings, our systematic review and meta-analyses do not support favorable effects of caseload midwifery regarding the risk of preterm birth or the risk of instrumental birth. Furthermore, the Cochrane review reported favorable effects of the intervention regarding their primary mortality outcome, which in addition to perinatal mortality also included fetal loss before 24 weeks [1]. Nevertheless, when perinatal mortality was included only, our results were consistent with the Cochrane review, showing no difference between the comparison groups. Methodological aspects may explain the partly divergent findings. In our meta-analyses, for instance, we only included RCTs without major risk of bias whereas the Cochrane review included all RCTs [1]. Furthermore, data used from one of the RCTs [21], in the meta-analysis of preterm birth in the Cochrane review, seem to be wrongly extracted. When the correct data are used instead (data not shown), the results regarding preterm birth are consistent with ours, i.e. no statistically significant difference. Finally, our comparison, where the same midwife usually provides antenatal care and checkup postnatally, also represents continuity to some extent.

Synthesizing available literature, we found that the only difference between caseload midwifery and standard care concerned the risk of caesarean section. This result was primarily based on studies performed in countries with a high incidence, illustrated, for instance, with the estimate of 25% caesarean section in the low-risk population included in the largest study [21]. With an overall incidence below 20% in 2021 [6], caesarean section is in general less common in Sweden than the worldwide average of 21% in 2018, and considerably lower than 25%, the average for countries in Northern Europe [26]. This may have implications for the applicability of the results. Indeed, the summarized absolute risk reduction of 4% units may neither be reasonable nor desirable in settings with a low incidence of caesarean sections; this intervention is also performed for medical reasons. Interestingly, our results of a significantly reduced incidence of caesarean section differ from the prior Cochrane review, where no difference was reported for midwife-led continuity models versus other models of care, with high certainty confidence in the evidence, and similar results in the subgroup analysis specifically focusing on caseload midwifery [1]. As elaborated upon above, differences in comparison groups as well as methodology may contribute to the divergent results.

Regarding perineal tear and bleeding, our meta-analyses show that there may be no difference between caseload midwifery and standard care. Regarding perineal tear irrespective of grade, however, it may be worth noting that there were only two RCTs available, too heterogeneous to be pooled, and that one of them significantly favored standard care [25].

Notably, the evidence regarding breastfeeding after discharge was inconclusive. Although one study provided data, the risk of bias was conspicuous due to very large loss-to-follow-up, with a skewed distribution between groups [25]. Future RCTs would be required to gain knowledge regarding the potential effect of the caseload model of care on breastfeeding.

For the important maternal outcomes HRQL and postpartum depression, no conclusions could be drawn from available literature. Neither could any conclusions be drawn regarding potential effects of caseload midwifery on health care experience/satisfaction and confidence. Furthermore, no study focused specifically on pregnant women with fear of birth, a subgroup that could benefit from further attention in future RCTs investigating the effects of caseload midwifery. Another subgroup that may deserve further attention, partly overlapping with fear of birth, could be women with previous trauma, including birth trauma.

Regarding feasibility, available literature shows that more than every second woman in a caseload model of care has their primary caseload midwife present at birth. With a caseload team of 12, nine in 10 women could be expected to have any of the team midwives present at birth, and the corresponding numbers with a team of six was eight in 10. These results could be of value for women entering the model, for informed expectations.

An important strength of this systematic review is that it, with a quantitative approach, provides a synthesis of currently available evidence regarding child and maternal outcomes for caseload midwifery compared with standard care, the latter defined as the same midwife usually providing care during pregnancy and postnatally but not during birth. Indeed, the fact that several studies were excluded as physicians provided much of the care illustrates the diversity of models of care worldwide. This may also have implications for the generalizability; our results may primarily be applicable in settings where midwives are the main care providers, and physicians if medical issues arise. Another strength of this review is the performance, being guided by an established HTA process and including thorough and transparent assessments. It could also be considered a strength that studies with major risk of bias were not included in our meta-analyses; this approach may increase the certainty of evidence.

Limitations of the present review include that few studies fulfilled our PICO. Furthermore, no RCTs at all could be identified for some outcomes, and for others, the certainty of evidence was very low and inconclusive. Another limitation is that we focused on quantifiable outcomes; an evidence synthesis of qualitative research could provide additional insights. The subgroup of women with fear of birth and/or previous birth trauma could be a subgroup of particular interest in such an evidence synthesis.

## Conclusions

This systematic review shows that there may be little or no difference between caseload midwifery and standard care regarding perinatal mortality and neonatal morbidity. Furthermore, the risk of preterm birth, as well as instrumental birth, is probably not affected. Regarding perineal tear, there may be no difference between the comparison groups. Caseload midwifery may reduce the incidence of caesarean section. No evidence is available regarding severe neonatal morbidity, and no conclusions can be drawn regarding breastfeeding after hospital discharge, maternal mortality, intensive care, HRQL, postpartum depression, and health care experience/satisfaction/confidence. As evidence regarding some critical and important child and maternal outcomes is lacking, and the certainty of evidence for others is very low or low, additional RCTs in relevant settings could add further insights regarding the potential of caseload midwifery model of care.

### Electronic supplementary material

Below is the link to the electronic supplementary material.

Supplementary Material 1

Supplementary Material 2

Supplementary Material 3

Supplementary Material 4

Supplementary Material 5

## Data Availability

The datasets supporting the conclusions of this article are included within the article and its additional file.

## References

[1] Sandall J, Soltani H, Gates S (2016). Midwife-led continuity models versus other models of care for childbearing women. Cochrane Database Syst Rev.

[2] Jepsen I, Juul S, Foureur MJ (2018). Labour outcomes in caseload midwifery and standard care: a register-based cohort study. BMC Pregnancy Childbirth.

[3] Wiegerinck MMJ, Eskes M, van der Post JAM (2020). Intrapartum and neonatal mortality in low-risk term women in midwife-led care and obstetrician-led care at the onset of labor: a national matched cohort study. Acta Obstet Gynecol Scand.

[4] NHS England. : Delivering midwifery continuity of Carer at full scale: Guidance on planning, implementation and monitoring 2021/22. PAR961 2021.

[5] Dawson K, McLachlan H, Newton M (2016). Implementing caseload midwifery: exploring the views of maternity managers in Australia - A national cross-sectional survey. Women Birth.

[6] Stephansson O, Petersson K, Björk C (2018). The swedish pregnancy Register - for quality of care improvement and research. Acta Obstet Gynecol Scand.

[7] Hanley A, Davis D, Kurz E (2021). Job satisfaction and sustainability of midwives working in caseload models of care: an integrative literature review. Women Birth.

[8] Perriman N, Davis DL, Ferguson S (2018). What women value in the midwifery continuity of care model: a systematic review with meta-synthesis. Midwifery.

[9] Page MJ, McKenzie JE, Bossuyt PM (2021). The PRISMA 2020 statement: an updated guideline for reporting systematic reviews. BMJ.

[10] Wassén L, Borgström Bolmsjö B, Eriksson M (2022). Nytta och risker med caseload midwifery: samma barnmorske-team genom graviditet, förlossning och eftervård [Benefits and risks of caseload midwifery: continuity of midwifery team during antenatal, intrapartal, and postnatal care].

[11] Granskningsmall RCT. [Checklist för RCTs] [https://www.vgregion.se/halsa-och-vard/vardgivarwebben/utveckling--uppfoljning/htacentrum/hjalpmedel-under-projektet/].

[12] Atkins D, Best D, Briss PA (2004). Grading quality of evidence and strength of recommendations. BMJ.

[13] Hildingsson I, Karlström A, Rubertsson C (2019). Women with fear of childbirth might benefit from having a known midwife during labour. Women Birth.

[14] Fernandez Turienzo C, Bick D, Briley AL (2020). Midwifery continuity of care versus standard maternity care for women at increased risk of preterm birth: a hybrid implementation-effectiveness, randomised controlled pilot trial in the UK. PLoS Med.

[15] Fernandez Turienzo C, Silverio SA, Coxon K (2021). Experiences of maternity care among women at increased risk of preterm birth receiving midwifery continuity of care compared to women receiving standard care: results from the POPPIE pilot trial. PLoS ONE.

[16] Forster DA, McLachlan HL, Davey MA (2016). Continuity of care by a primary midwife (caseload midwifery) increases women’s satisfaction with antenatal, intrapartum and postpartum care: results from the COSMOS randomised controlled trial. BMC Pregnancy Childbirth.

[17] Homer CS, Davis GK, Brodie PM (2001). Collaboration in maternity care: a randomised controlled trial comparing community-based continuity of care with standard hospital care. BJOG.

[18] Homer CS, Davis GK, Cooke M (2002). Women’s experiences of continuity of midwifery care in a randomised controlled trial in Australia. Midwifery.

[19] Homer CSE, Davis DL, Mollart L (2021). Midwifery continuity of care and vaginal birth after caesarean section: a randomised controlled trial. Women Birth.

[20] Marks MN, Siddle K, Warwick C (2003). Can we prevent postnatal depression? A randomized controlled trial to assess the effect of continuity of midwifery care on rates of postnatal depression in high-risk women. J Matern Fetal Neonatal Med.

[21] McLachlan HL, Forster DA, Davey MA (2012). Effects of continuity of care by a primary midwife (caseload midwifery) on caesarean section rates in women of low obstetric risk: the COSMOS randomised controlled trial. BJOG.

[22] McLachlan HL, Forster DA, Davey MA (2016). The effect of primary midwife-led care on women’s experience of childbirth: results from the COSMOS randomised controlled trial. BJOG.

[23] Morrison J, Neale L, Taylor R (2002). Caring for pregnant women with diabetes. Br J Midwifery.

[24] North Staffordshire Changing Childbirth Research Team (2000). A randomised study of midwifery caseload care and traditional ‘shared-care’. Midwifery.

[25] Tracy SK, Hartz DL, Tracy MB (2013). Caseload midwifery care versus standard maternity care for women of any risk: M@NGO, a randomised controlled trial. Lancet.

[26] Betran AP, Ye J, Moller AB et al. Trends and projections of caesarean section rates: global and regional estimates. BMJ Glob Health 2021;6. 10.1136/bmjgh-2021-005671 PMC8208001 34130991

