# Supplementary material for: Child and maternal benefits and risks of caseload midwifery – a systematic review and meta-analysis
Source: BMC Pregnancy Childbirth. 2023 Sep 15;23:663. doi: 10.1186/s12884-023-05967-x (PMC10504769; doi:10.1186/s12884-023-05967-x)
Supplement: Supplementary file 1 — Supplementary Material 1 [file 12884_2023_5967_MOESM1_ESM.docx]

**Search strategies**

**Database:** Ovid MEDLINE(R) ALL

**Date:** 4 Nov 2021
**No. of results:** 1543

| **#** | **Searches** | **Results** |
| --- | --- | --- |
| 1 | (midwi$ adj3 (continu$ or caseload$)).mp. | 500 |
| 2 | (midwi$ adj2 team$).mp. | 159 |
| 3 | (midwi$ adj (model$ or led or manag$ or group or one-to-one)).mp. | 1022 |
| 4 | lead maternity carer.mp. | 27 |
| 5 | primary care midwives.mp. | 49 |
| 6 | community midwife.mp. | 84 |
| 7 | 1 or 2 or 3 or 4 or 5 or 6 | 1623 |
| 8 | (comment or editorial).pt. | 1337304 |
| 9 | 7 not 8 | 1574 |
| **10** | **limit 9 to (danish or english or norwegian or swedish)** | **1543** |

**Database:** Embase 1974 to 2017 January 09 (Ovid)

**Date:** 4 Nov 2021

**No. of results:** 1541

| **#** | **Searches** | **Results** |
| --- | --- | --- |
| 1 | (midwi$ adj3 (continu$ or caseload$)).mp. | 587 |
| 2 | (midwi$ adj2 team$).mp. | 250 |
| 3 | (midwi$ adj (model$ or led or manag$ or group or one-to-one)).mp. | 1266 |
| 4 | lead maternity carer.mp. | 30 |
| 5 | primary care midwives.mp. | 57 |
| 6 | community midwife.mp. | 118 |
| 7 | 1 or 2 or 3 or 4 or 5 or 6 | 2042 |
| 8 | editorial.pt. | 707315 |
| 9 | 7 not 8 | 2031 |
| 10 | limit 9 to (embase or medline) | 1580 |
| **11** | **limit 10 to (danish or english or norwegian or swedish)** | **1541** |

**Database:** The Cochrane Library
**Date:** 4 Nov 2021
**No of results:** 201 ref

*Cochrane reviews: 10
Cochrane protocols: 0
Trials: 189
Editorials: 0
Special collections: 0
Clinical answers: 2*

| **ID** | **Search** | **Hits** |
| --- | --- | --- |
| #1 | ((midwi* NEAR/3 (continu* or caseload*))):ti,ab,kw (Word variations have been searched) | 85 |
| #2 | ((midwi* NEAR/2 team*)):ti,ab,kw (Word variations have been searched) | 33 |
| #3 | ((midwi* NEXT (model* or led or manag* or group or one-to-one))):ti,ab,kw (Word variations have been searched) | 179 |
| #4 | ("lead maternity carer"):ti,ab,kw (Word variations have been searched) | 2 |
| #5 | ("primary care midwives"):ti,ab,kw (Word variations have been searched) | 5 |
| #6 | ("community midwife"):ti,ab,kw (Word variations have been searched) | 19 |
| #7 | #1 OR #2 OR #3 OR #4 OR #5 OR #6 | 276 |
| #8 | (clinicaltrials OR trialsearch):so | 381,884 |
| #9 | (conference abstract):pt | 184,800 |
| #10 | #8 OR #9 | 566,684 |
| **#11** | **#7 NOT #10** | **201** |

___________________________________________________________________________

**Database:** CINAHL (EBSCOhost)

**Date:** 4 Nov 2021

**No. of results:** 1,965

| **#** | **Query** | **Results** |
| --- | --- | --- |
| **S12** | **S1 OR S2 OR S8 OR S9 OR S10 OR S11**  **Limiters - Publication Type: Case Study, Clinical Trial, Corrected Article, Journal Article, Letter, Meta Analysis, Meta Synthesis, Nursing Interventions, Practice Guidelines, Randomized Controlled Trial, Research, Review, Systematic Review; Language: Danish, English, Norwegian, Swedish** | **1,965** |
| S11 | "community midwife" | 112 |
| S10 | "primary care midwives" | 47 |
| S9 | "lead maternity carer" | 49 |
| S8 | S3 OR S4 OR S5 OR S6 OR S7 | 1,345 |
| S7 | "midwi* one-to-one" OR "one-to-one midwi*" | 56 |
| S6 | "midwi* group" | 140 |
| S5 | "midwi* manag*" | 231 |
| S4 | "midwi* led" | 792 |
| S3 | "midwi* model" | 175 |
| S2 | (midwi* N2 team*) | 253 |
| S1 | (midwi* N3 (continu* or caseload*)) | 834 |

The web-sites of **SBU** and **Folkehelseinstituttet** were visited 4 Nov 2021

Nothing relevant to the question at issue was found

**Reference lists**

A comprehensive review of reference lists and other sources brought 38 new records

**Ongoing trials**

A search was performed in Clinicaltrials.gov and WHO ICTRP (March 28^th^, 2022) using the terms ((midwifery OR midwife OR midwives) AND (caseload OR case-load OR continuity OR one-to-one OR team OR model OR models or led)) OR "lead maternity carer" OR "primary care midwives" OR "community midwife".
