# Supplementary material for: Child and maternal benefits and risks of caseload midwifery – a systematic review and meta-analysis
Source: BMC Pregnancy Childbirth. 2023 Sep 15;23:663. doi: 10.1186/s12884-023-05967-x (PMC10504769; doi:10.1186/s12884-023-05967-x)
Supplement: Supplementary file 2 — Supplementary Material 2 [file 12884_2023_5967_MOESM2_ESM.docx]

**Supplemental Table 1** Studies excluded after full-text reading, as well as the reason for excluding them.

(For full citations, see Additional file 5.)

| **Author Year** | **Reason for exclusion** |
| --- | --- |
| Allen 2015 | Wrong study design: non-randomized study |
| Allen 2016 | Wrong study design: non-randomized study |
| Allen 2017 | Wrong study design: qualitative analysis |
| Allen 2019 | Wrong outcome: a study of perception of care during pregnancy, not during delivery or postnatally |
| Allen 2020 | Wrong study design: qualitative analysis |
| Attanasio 2020 | Wrong outcome: health economics |
| Bagheri 2021 | Wrong comparison: insufficient description, most likely physician-led care |
| Bai 2008 | Wrong study design: non-randomized study |
| Bartuseviciene 2018 | Wrong study design: non-randomized study |
| Beckmann 2012 | Wrong study design: non-randomized study |
| Begley 2011 | Wrong intervention: explained as not the same continuity as in caseload |
| Benatar 2013 | Wrong study design: non-randomized study |
| Bernitz 2011 | Wrong intervention: only in the delivery ward |
| Bernitz 2012 | Wrong intervention: only in the delivery ward |
| Biro 1991 | Wrong comparison: no control group |
| Biro 2000 | Wrong comparison: most visits to a physician |
| Biro 2003 | Wrong comparison: most visits to a physician |
| Bodner-Adler 2017 | Wrong intervention: only in the delivery ward |
| Butler 1993 | Wrong intervention and comparison: only delivery, pregnancy and postnatal care not included |
| Callander 2021 | Wrong outcome: health economics |
| Chambliss 1992 | Wrong intervention: only delivery ward |
| Chunyi 2013 | Wrong comparison: obstetrician antenatally |
| COAG Health Council, 2019 | Wrong publication type: report |
| Dale 2010 | Wrong publication type: narrative report of experiences in one department |
| Dante 2016 | Wrong intervention: no antenatal part, not caseload |
| Davey 2013 | No outcome of interest: study of time at delivery ward before childbirth |
| de Jonge 2015 | Wrong intervention: only in the delivery ward |
| de Wolff 2021 | Wrong intervention: no known midwife during delivery |
| Donnellan-Fernandez 2018 | Wrong outcome: health economics, review |
| Durst 2016 | Wrong study design: non-randomized study |
| Eide 2009 | Wrong intervention: no known midwife during delivery |
| Farquhar 2000 | Wrong study design: non-randomized study |
| Farry 2019 | Wrong intervention: midwife-led delivery wards, no caseload. Wrong comparison: only university hospitals. |
| Fawsitt 2017 | Wrong outcome: health economics |
| Flint 1989 | Wrong comparison: not described |
| Flood 2012 | Wrong publication type: abstract for poster |
| Forti 2015 | Wrong publication type: abstract for poster |
| Gidaszewski 2019 | Wrong study design: non-randomized study |
| Giles 1992 | Wrong intervention: no delivery, only antenatal care |
| Gottvall 2011 | Wrong study design: non-randomized study |
| Gutteridge 2015 | Wrong intervention: birth centre with homelike environment, not continuity of care |
| Hailemeskel 2021 | Wrong comparison: no control group |
| Hanley 2021 | Wrong study design: systematic Review. Wrong outcome: midwifery-related outcomes |
| Harvey 1996 | Wrong comparison: only obstetrician/family doctor |
| Harvey 2002 | Wrong comparison: only physicians |
| Hicks 2003 | Wrong comparison: caseload is a part of the comparison |
| Hildingsson 2014 | Wrong intervention: only antenatal care |
| Hildingsson 2019 | Wrong study design: non-randomized study |
| Hildingsson 2020a | Wrong study design: non-randomized study |
| Hildingsson 2020b | Wrong intervention: on call duty rather than caseload |
| Hildingsson 2021a | Wrong study design: non-randomized study |
| Hildingsson 2021b | Wrong study design: non-randomized study |
| Hildingsson 2021c | Wrong intervention: focus on women’s experience of antenatal care only |
| Holmes 1996 | Wrong intervention: a model description, no comparison |
| Homer 2000 | Wrong intervention: only antenatal care |
| Homer 2001b | Wrong outcome: health economics |
| Huber 2006 | Wrong study design: non-randomized study |
| Hundley 1994 | Wrong intervention: only in the delivery ward |
| Hundley 1995a | Wrong population: focus on the midwife |
| Hundley 1995b | Wrong intervention: only in the delivery ward |
| Hundley 1997 | Wrong intervention: only in the delivery ward |
| Hunter 2008 | Wrong publication type: commentary, review |
| Huynh 2014 | Wrong comparison: only physicians. Intervention unclearly described. |
| Iida 2014 | Wrong study design: non-randomized study |
| Isaline 2019 | Wrong outcome: health economics. Wrong study design: non-randomized study. |
| Jepsen 2018 | Wrong study design: non-randomized study |
| Jiang 2018 | Wrong intervention: only in the delivery ward |
| Kataoka 2018 | Wrong intervention: only in the delivery ward |
| Kenny 1994 | Wrong comparison: antenatal care given by physicians only |
| Kenny 2015 | Wrong intervention: explained as not the same continuity as in caseload (compare Begley 2011) Wrong outcome: health economics |
| Koto 2019 | Wrong study design: non-randomized study. Wrong outcome: health economics |
| Law 1999 | Wrong intervention: only in the delivery ward |
| Lawton 2013 | Wrong comparison: physicians only. Wrong intervention: probably not continuity of care |
| Lewis 2016 | Wrong study design: non-randomized study |
| Li 2014 | Wrong intervention: no continuity of care. Wrong study design: non-randomized study |
| McCormick 2021 | Wrong publication type: review |
| McGinley 1995 | Wrong publication type: description of a clinic, no patient data |
| Mortensen 2018 | Wrong intervention: including only antenatal and postnatal care, not delivery |
| Mortensen 2019 | Wrong study design: non-randomized study |
| Offerhaus 2014 | Article withdrawn, replaced by Offerhaus 2015 |
| Offerhaus 2015 | Wrong intervention: unclear if continuity of care. Wrong comparison: no control group. Wrong study design: case series |
| O'Leary 2020 | Wrong intervention: only in the delivery ward |
| Pace 2021 | Wrong study design: systematic review of qualitative studies |
| Page 2001 | Wrong study design: non-randomized study |
| Permezel 2015 | Wrong intervention and comparison: 4 models, no clear caseload or standard care could be identified. |
| Perriman 2018 | Wrong study design: systematic review of qualitative studies |
| Poskiene 2021 | Wrong study design: non-randomized study |
| Relph 2020 | Wrong publication type: general review. Wrong intervention: many different interventions in UK maternity care. |
| Ricchi 2019 | Wrong publication type: review |
| Rowley 1995 | Wrong comparison: most visits to a physician |
| Ryan 2013 | Wrong study design: systematic review, health economics |
| Sandall 2016 | Wrong study design: systematic review |
| Seijmonsbergen-Schermers 2020 | Wrong intervention and comparison: study of regional variations |
| Shields 1998 | Wrong intervention: not current model of caseload. Wrong comparison: standard care with unclear part of general physicians and obstetricians |
| Shields 1999 | Wrong intervention: known midwife rather than caseload. Wrong comparison: unknown midwife |
| Spurgeon 2001 | Wrong study design: non-randomized study |
| Sutton 2002 | Wrong study design: non-randomized study |
| Suzuki 2014 | Wrong intervention: only in the delivery ward, no continuity |
| Suzuki 2016 | Wrong intervention: only in the delivery ward, no continuity |
| Symon 2015 | Wrong intervention: only antenatal care |
| Talukdar 2021 | Wrong study design: scoping review |
| Thiessen 2016 | Wrong intervention: only during delivery |
| Tracy 2014 | Wrong comparison: unclear description. Wrong outcome: health economics |
| Tucker 2000 | Wrong study design: non-randomized study |
| Turnbull 1995 | Wrong publication type: description of a clinic, no patient data |
| Turnbull 1996 | Wrong intervention: not current model of caseload: women were referred to the obstetric medical team when there was deviation from normal. Wrong comparison: standard care with unclear part of general physicians and obstetricians |
| Turnbull 1999 | Wrong intervention: not current model of caseload. Wrong comparison: standard care with unclear part of general physicians and obstetricians |
| Waldenström 1993 | Wrong intervention: alternative birth center (ABC) without fetal monitoring and pharmacological pain treatment |
| Waldenström 1994a | Wrong intervention: alternative birth center (ABC) without fetal monitoring and pharmacological pain treatment |
| Waldenström 1994b | Wrong intervention: alternative birth center (ABC) without fetal monitoring and pharmacological pain treatment |
| Waldenström 1997a | Wrong intervention: alternative birth center (ABC) without fetal monitoring and pharmacological pain treatment |
| Waldenström 1997b | Wrong intervention: alternative birth center (ABC) without fetal monitoring and pharmacological pain treatment |
| Waldenström 2000 | Wrong comparison: high proportion of visits to a physician |
| Waldenström 2001 | Wrong comparison: high proportion of visits to a physician. Substudy to Waldenström 2000. |
| Wernham 2016 | Wrong comparison. Physician-led care model |
| Wiegerinck 2015 | Wrong intervention: no continuity of care |
| Wiegerinck 2018 | Wrong intervention: no continuity of care |
| Wiegerinck 2020 | Wrong intervention: no continuity of care |
| Wilson 2010 | Wrong intervention and comparison: choice between midwife or obstetrician during delivery |
| Young 1997a | Wrong intervention: only antenatal care |
| Young 1997b | Wrong outcome: health economics |
| Zhang 2016 | Wrong intervention and comparison: only during labour |
| Zhang 2021 | Wrong intervention and comparison: only delivery |
