# Supplementary material for: Child and maternal benefits and risks of caseload midwifery – a systematic review and meta-analysis
Source: BMC Pregnancy Childbirth. 2023 Sep 15;23:663. doi: 10.1186/s12884-023-05967-x (PMC10504769; doi:10.1186/s12884-023-05967-x)
Supplement: Supplementary file 3 — Supplementary Material 3 [file 12884_2023_5967_MOESM3_ESM.docx]

**Supplemental Table 2** Assessments of directness and risk of bias of included studies being categorized as having no/minor (+), some (?) or major (-) problems.

(For full citations, see Additional file 5.)

| **Author Year**  **Country**  Acronym | **Problems contributing to the assessment** | | | |
| --- | --- | --- | --- | --- |
|  | **Directness** | | **Risk of bias** | |
| Fernandez Turienzo 2020  England  POPPIE | ? | P: High risk of preterm birth, low socio-economic status | ? | **Selection bias:** Skewed distribution of some baseline characteristics, adjustments in the statistical analysis  **Performance bias:** Open trial  **Detection bias:** Primary outcome sensitive to assessment (initiation of and adequate timing of interventions to manage risk of preterm birth  **COI bias:** Authors report fees from the industry, but probably unrelated to caseload midwifery |
| Fernandez Turienzo 2021  England  POPPIE | ? | P: High risk of preterm birth, low socio-economic status | - | **Selection bias:** Skewed distribution of some baseline characteristics, adjustments in the statistical analysis  **Performance bias:** Open trial  **Detection bias:** Primary outcome sensitive to assessment (PROMIS-10)  **Attrition bias:** 90 (60.4%) vs 76 (49.4%) response rate to questionnaire  **COI bias:** Authors report fees from the industry, but probably unrelated to caseload midwifery |
| Forster 2016  Australia  COSMOS | ? | P: Low risk population with anticipated CS rate of 25%  C: Antenatal visits to midwifes (78%), others mainly to GPs  O: Stated outcome Parental satisfaction is not in complete accordance with the reported outcome | - | **Performance bias:** Open trial  **Detection bias:** Primary outcome sensitive to assessment  **Attrition bias**: 172 (15%) vs 328 (28%) randomized women were not included in the analysis |
| Homer 2001a  Australia  STOMP | ?/- | P: Recruitment 1997-1998 (CTG surveillance during labor for half of the women, hospital stay 5 days post-partum)  C: 90 women (16%) had antenatal visits to GPs | ? | **Selection bias:** Skewed distribution of some baseline characteristics, no adjustments in the statistical analysis  **Performance bias:** Open trial  **Selective reporting:** No trial protocol |
| Homer 2002  Australia  STOMP | ? | P: As above. This trial contributes with the outcome compliance in the intervention arm | ? | As above |
| Homer 2021  Australia | ?/- | P: Previous CS  C: Rotating roaster more common than in Sweden | - | **Performance bias:** Open trial  52.9% in C knew the midwife assisting during delivery  **Detection bias:** Primary outcome sensitive to assessment (planned vaginal delivery at gestational week 36)  **Selective reporting:** No trial protocol  **COI bias:** First author is editor-in-chief for the journal in which the article is published |
| Marks 2003  England | - | P: Only women with previous depression  O: Unclear diagnosis for post-partum depression | ? | **Selection bias:** Randomization procedure not described, minimization after inclusion of 70 to counteract skewed distribution  **Performance bias:** Open trial. 2 vs 6 did not receive allocated treatment  **Detection bias:** Unclear reporting on the use of the instruments for psychiatric morbidity  **Attrition bias:** 4 (9%) vs 7 (14%) were not included in the analysis  **Selective reporting:** No trial protocol, unclear reporting of scales |
| McLachlan 2012  Australia  COSMOS | ? | P: Low risk population with anticipated CS rate of 25%  C: Antenatal visits to midwifes (78%), others mainly to GPs | + | **Performance bias:** Open trial, less important for the primary outcome CS |
| McLachlan 2016  Australia  COSMOS | ? | P: Low risk population with anticipated CS rate of 25%  C: Antenatal visits to midwifes (78%), others mainly to GPs | - | **Performance bias:** Open trial  **Detection bias:** Primary outcome sensitive to assessment  **Attrition bias**:172 (15%) vs 328 (28%) randomized women were not included in the analysis |
| Morrison 2002  New Zealand | - | P: Pregnant women with diabetes. Recruitment 1997-1999 (longer hospital stay than at present)  I: Only one team, three midwives | - | **Selection bias:** Unclear randomization  **Performance bias:** Open trial  **Detection bias:** Hospital stay reported in days (unprecise measure)  **Attrition bias:** Outcome-dependent. 14 (10%) vs 17 (12%) in questionnaire outcomes  **Selective reporting:** No trial protocol  **COI bias:** Not reported |
| North Staffordshire 2000  England | - | P: Cluster not described. Inclusion period not specified  C: Standard care not described, 10% assisted at birth by known midwife | - | **Selection bias:** Cluster randomization  **Performance bias:** Open trial  **Detection bias:** Analysis per individual despite cluster randomization. Perineal tears not reported according to modern standard  **Attrition bias:** Numbers are unclear  **Selective reporting:** No trial protocol  **COI:** Not reported |
| Tracy 2013  Australia  (M@NGO) | ? | P: 27% of screened subjects were randomized, unclear reporting of non-randomized  C: Unclear if standard care in the trial corresponds to Swedish standard care (the intention is that the woman meets the same midwife during antenatal care). In the trial, the women may meet a different midwife at every visit | ? | **Performance bias:** Open trial. Compliance to allocated intervention differed between groups  (19 versus 65 crossed over)  **Detection bias**: Outcome-dependent. The primary maternal outcome CS is not sensitive. The primary neonatal outcome is sensitive (Apgar ≤7 at 5 minutes)  **Attrition bias:** Outcome-dependent. CS is not sensitive. Outcome with large losses in relation to number of events is sensitive to attrition bias. The outcome breastfeeding has very large losses with a skewed distribution between groups |

COI = conflict of interest, COSMOS = COmparing Standard Maternity care with One-to-one midwifery Support, CS = caesarean section, CTG = cardiotocography, GP = general practitioner, M@NGO = Midwives @ New Group practice Options, POPPIE = Pilot study Of midwifery Practice in Preterm birth Including women’s Experiences, STOMP = St George Outreach Maternity Project
