# Supplementary material for: Child and maternal benefits and risks of caseload midwifery – a systematic review and meta-analysis
Source: BMC Pregnancy Childbirth. 2023 Sep 15;23:663. doi: 10.1186/s12884-023-05967-x (PMC10504769; doi:10.1186/s12884-023-05967-x)
Supplement: Supplementary file 4 — Supplementary Material 4 [file 12884_2023_5967_MOESM4_ESM.docx]

**Supplemental Table 3** GRADE assessments, reasons for downgrading the certainty of evidence per outcome, across studies.

|  | | **Study limitations** | **Consistency** | **Directness** | **Precision** | **Conclusion** | **Certainty of evidence** |
| --- | --- | --- | --- | --- | --- | --- | --- |
| *Child outcomes* | |  |  |  |  |  |  |
| Perinatal mortality | | Some limitations | No serious inconsistency | Some uncertainty | Serious imprecision | Little/no difference | ⊕⊕🌕🌕 |
| Perinatal morbidity | Severe morbidity |  |  |  |  |  | - |
|  | Apgar ≤4 at 5 min |  |  |  |  |  | - |
|  | Apgar ≤7 at 5 min | Some limitations | No serious inconsistency | Some uncertainty | Uncertain precision | Little/no difference | ⊕⊕⊕🌕 |
|  | NICU | Some limitations | No serious inconsistency | Some uncertainty | Serious imprecision | Little/no difference | ⊕⊕🌕🌕 |
| Breastfeeding | | Very serious limitations | NA | Some uncertainty | Uncertain precision | NA | ⊕🌕🌕🌕 |
| *Maternal outcomes* | |  |  |  |  |  |  |
| Mortality | | Some limitations | No serious inconsistency | Some uncertainty | Very serious imprecision | NA | ⊕🌕🌕🌕 |
| Intensive care | | Some limitations | NA | Some uncertainty | Very serious imprecision | NA | ⊕🌕🌕🌕 |
| HRQL | | Very serious limitations | NA | Serious indirectness | Uncertain precision | NA | ⊕🌕🌕🌕 |
| Perineal tear | I-IV | Some limitation | Serious inconsistency | Some uncertainty | Uncertain precision | Little/no difference | ⊕⊕🌕🌕 |
|  | III-IV | Some limitations | No serious inconsistency | Some uncertainty | Serious imprecision | Little/no difference | ⊕⊕🌕🌕 |
| Bleeding (>1000 ml) | | Some limitations | No serious inconsistency | Some uncertainty | Serious imprecision | Little/no difference | ⊕⊕🌕🌕 |
| Caesarian section | All | Some limitations | No serious inconsistency | Very serious indirectness | No imprecision | I: reduced frequency | ⊕⊕🌕🌕 |
|  | Acute | Some limitations | Some inconsistency | Serious indirectness | No imprecision | Little/no difference | ⊕⊕🌕🌕 |
| Instrumental birth | | Some limitations | No serious inconsistency | Serious indirectness | No imprecision | Little/no difference | ⊕⊕⊕🌕 |
| Postpartum depression | | Some limitations | NA | Very serious indirectness | Serious imprecision | NA | ⊕🌕🌕🌕 |
| Preterm birth | | Some limitations | Some inconsistency | Some uncertainty | Uncertain precision | Little/no difference | ⊕⊕⊕🌕 |
| Health care experience/satisfaction/confidence | | Very serious limitations | Some inconsistency | Some uncertainty | Uncertain precision | NA | ⊕🌕🌕🌕 |

HRQL = health-related quality of life, I = intervention caseload midwifery, NA = not applicable, NICU = neonatal intensive care un
