# Supplementary material for: Child and maternal benefits and risks of caseload midwifery – a systematic review and meta-analysis
Source: BMC Pregnancy Childbirth. 2023 Sep 15;23:663. doi: 10.1186/s12884-023-05967-x (PMC10504769; doi:10.1186/s12884-023-05967-x)
Supplement: Supplementary file 5 — Supplementary Material 5 [file 12884_2023_5967_MOESM5_ESM.docx]

**Full references**

Included studies

1. Fernandez Turienzo C, Bick D, Briley AL, et al. Midwifery continuity of care versus standard maternity care for women at increased risk of preterm birth: A hybrid implementation-effectiveness, randomised controlled pilot trial in the UK. PLoS Med. 2020;17:e1003350.
2. Fernandez Turienzo C, Silverio SA, Coxon K, et al. Experiences of maternity care among women at increased risk of preterm birth receiving midwifery continuity of care compared to women receiving standard care: Results from the POPPIE pilot trial. PLoS One. 2021;16:e0248588.
3. Forster DA, McLachlan HL, Davey MA, et al. Continuity of care by a primary midwife (caseload midwifery) increases women's satisfaction with antenatal, intrapartum and postpartum care: results from the COSMOS randomised controlled trial. BMC Pregnancy Childbirth. 2016;16:28.
4. Homer CS, Davis GK, Brodie PM, et al. Collaboration in maternity care: a randomised controlled trial comparing community-based continuity of care with standard hospital care. BJOG. 2001;108:16-22.
5. Homer CS, Davis GK, Cooke M, Barclay LM. Women's experiences of continuity of midwifery care in a randomised controlled trial in Australia. Midwifery. 2002;18:102-112.
6. Homer CSE, Davis DL, Mollart L, et al. Midwifery continuity of care and vaginal birth after caesarean section: A randomised controlled trial. Women Birth. 2021;05:05.
7. Marks MN, Siddle K, Warwick C. Can we prevent postnatal depression? A randomized controlled trial to assess the effect of continuity of midwifery care on rates of postnatal depression in high-risk women. J Matern Fetal Neonatal Med. 2003;13:119-127.
8. McLachlan HL, Forster DA, Davey MA, et al. The effect of primary midwife-led care on women's experience of childbirth: results from the COSMOS randomised controlled trial. BJOG. 2016;123:465-474.
9. McLachlan HL, Forster DA, Davey MA, et al. Effects of continuity of care by a primary midwife (caseload midwifery) on caesarean section rates in women of low obstetric risk: the COSMOS randomised controlled trial. BJOG. 2012;119:1483-1492.
10. Morrison J, Neale L, Taylor R, McGowan L. Caring for pregnant women with diabetes. Br J Midwifery. 2002;10:434-439.
11. North Staffordshire Changing Childbirth Research Team. A randomised study of midwifery caseload care and traditional 'shared-care'. Midwifery. 2000;16:295-302.
12. Tracy SK, Hartz DL, Tracy MB, et al. Caseload midwifery care versus standard maternity care for women of any risk: M@NGO, a randomised controlled trial. Lancet. 2013;382:1723-1732.

Excluded studies

1. Allen J, Gibbons K, Beckmann M, Tracy M, Stapleton H, Kildea S. Does model of maternity care make a difference to birth outcomes for young women? A retrospective cohort study. Int J Nurs Stud. 2015;52:1332-1342.
2. Allen J, Jenkinson B, Tracy SK, Hartz DL, Tracy M, Kildea S. Women's unmet needs in early labour: Qualitative analysis of free-text survey responses in the M@NGO trial of caseload midwifery. Midwifery. 2020;88:102751.
3. Allen J, Kildea S, Hartz DL, Tracy M, Tracy S. The motivation and capacity to go 'above and beyond': Qualitative analysis of free-text survey responses in the M@NGO randomised controlled trial of caseload midwifery. Midwifery. 2017;50:148-156.
4. Allen J, Kildea S, Stapleton H. How optimal caseload midwifery can modify predictors for preterm birth in young women: Integrated findings from a mixed methods study. Midwifery. 2016;41:30-38.
5. Allen J, Kildea S, Tracy MB, Hartz DL, Welsh AW, Tracy SK. The impact of caseload midwifery, compared with standard care, on women's perceptions of antenatal care quality: Survey results from the M@NGO randomized controlled trial for women of any risk. Birth. 2019;46:439-449.
6. Attanasio LB, Alarid-Escudero F, Kozhimannil KB. Midwife-led care and obstetrician-led care for low-risk pregnancies: A cost comparison. Birth. 2020;47:57-66.
7. Bagheri A, Simbar M, Samimi M, Nahidi F, Alavimajd H, Sadat Z. Comparing the Implications of Midwifery-Led Care and Standard Model on Maternal and Neonatal Outcomes during Pregnancy, Childbirth and Postpartum. JMRH. 2021;9:1‐10.
8. Bai J, Gyaneshwar R, Bauman A. Models of antenatal care and obstetric outcomes in Sydney South West. Aust N Z J Obstet Gynaecol. 2008;48:454-461.
9. Bartuseviciene E, Kacerauskiene J, Bartusevicius A, et al. Comparison of midwife-led and obstetrician-led care in Lithuania: A retrospective cohort study. Midwifery. 2018;65:67-71.
10. Beckmann M, Kildea S, Gibbons K. Midwifery group practice and mode of birth. Women Birth. 2012;25:187-193.
11. Begley C, Devane D, Clarke M, et al. Comparison of midwife-led and consultant-led care of healthy women at low risk of childbirth complications in the Republic of Ireland: a randomised trial. BMC Pregnancy Childbirth. 2011;11:85.
12. Benatar S, Garrett AB, Howell E, Palmer A. Midwifery care at a freestanding birth center: a safe and effective alternative to conventional maternity care. Health Serv Res. 2013;48:1750-1768.
13. Bernitz S, Aas E, Oian P. Economic evaluation of birth care in low-risk women. A comparison between a midwife-led birth unit and a standard obstetric unit within the same hospital in Norway. A randomised controlled trial. Midwifery. 2012;28:591-599.
14. Bernitz S, Rolland R, Blix E, Jacobsen M, Sjoborg K, Oian P. Is the operative delivery rate in low-risk women dependent on the level of birth care? A randomised controlled trial. BJOG. 2011;118:1357-1364.
15. Biro M, Lumley J. The safety of team midwifery: the first decade of the Monash Birth Centre. Med J Aust. 1991;155:478-480.
16. Biro MA, Waldenstrom U, Brown S, Pannifex JH. Satisfaction with team midwifery care for low- and high-risk women: a randomized controlled trial. Birth. 2003;30:1-10.
17. Biro MA, Waldenstrom U, Pannifex JH. Team midwifery care in a tertiary level obstetric service: a randomized controlled trial. Birth. 2000;27:168-173.
18. Bodner-Adler B, Kimberger O, Griebaum J, Husslein P, Bodner K. A ten-year study of midwife-led care at an Austrian tertiary care center: a retrospective analysis with special consideration of perineal trauma. BMC Pregnancy Childbirth. 2017;17:357.
19. Butler J, Abrams B, Parker J, Roberts JM, Laros RK, Jr. Supportive nurse-midwife care is associated with a reduced incidence of cesarean section. AJOG. 1993;168:1407-1413.
20. Callander EJ, Slavin V, Gamble J, Creedy DK, Brittain H. Cost-effectiveness of public caseload midwifery compared to standard care in an Australian setting: a pragmatic analysis to inform service delivery. Int J Qual Health Care. 2021;33:28.
21. Chambliss LR, Daly C, Medearis AL, Ames M, Kayne M, Paul R. The role of selection bias in comparing cesarean birth rates between physician and midwifery management. Obstet Gynecol. 1992;80:161-165.
22. Chunyi G, Xiaodan W, Yan D, Xinli Z, Zheng Z. The effectiveness of a Chinese midwives' antenatal clinic service on childbirth outcomes for primipare: A randomised controlled trial. Int J Nurs Stud. 2013;50:1689-1697.
23. Council CH. Woman-centred care: Strategic directions for Australian maternity services. 2019.
24. Dale A. Caseload midwifery: the Weston Shore experience. Midwifery Matters. 2010:6-7.
25. Dante G, Neri I, Bruno R, Salvioli C, Facchinetti F. Perinatal and maternal outcomes in a midwife-led centre in Italy: a comparison with standard hospital assistance. Minerva Ginecol. 2016;68:237-242.
26. Davey MA, McLachlan HL, Forster D, Flood M. Influence of timing of admission in labour and management of labour on method of birth: results from a randomised controlled trial of caseload midwifery (COSMOS trial). Midwifery. 2013;29:1297-1302.
27. de Jonge A, Mesman JA, Mannien J, et al. Severe Adverse Maternal Outcomes among Women in Midwife-Led versus Obstetrician-Led Care at the Onset of Labour in the Netherlands: A Nationwide Cohort Study. PLoS One. 2015;10:e0126266.
28. de Wolff MG, Midtgaard J, Johansen M, et al. Effects of a Midwife-Coordinated Maternity Care Intervention (ChroPreg) vs. Standard Care in Pregnant Women with Chronic Medical Conditions: Results from a Randomized Controlled Trial. Int J Environ Res Public Health. 2021;18:25.
29. Donnellan-Fernandez RE, Creedy DK, Callander EJ. Cost-effectiveness of continuity of midwifery care for women with complex pregnancy: a structured review of the literature. Health Econ Rev. 2018;8:32.
30. Durst M, Rolfe M, Longman J, et al. Local birthing services for rural women: Adaptation of a rural New South Wales maternity service. Aust J Rural Health. 2016;24:385-391.
31. Eide BI, Nilsen AB, Rasmussen S. Births in two different delivery units in the same clinic--a prospective study of healthy primiparous women. BMC Pregnancy Childbirth. 2009;9:25.
32. Farquhar M, Camilleri-Ferrante C, Todd C. Continuity of care in maternity services: women's views of one team midwifery scheme. Midwifery. 2000;16:35-47.
33. Farry A, McAra-CouperB J, Weldon MC, Clemons J. Comparing perinatal outcomes for healthy pregnant women presenting at primary and tertiary settings in South Auckland: A retrospective cohort study. NZCOM. 2019:5-13.
34. Fawsitt CG, Bourke J, Murphy A, et al. A Cost-Benefit Analysis of Two Alternative Models of Maternity Care in Ireland. Appl Health Econ Health Policy. 2017;15:785-794.
35. Flint C, Poulengeris P, Grant A. The 'Know Your Midwife' scheme--a randomised trial of continuity of care by a team of midwives. Midwifery. 1989;5:11-16.
36. Flood M, Forster DA, Davey MA, McLachlan HL. Serious adverse event monitoring in a RCT of caseload midwifery (COSMOS). J Paediatr Child Health. 2012;48:113.
37. Forti A, Kildea S, Stapleton H. Intrapartum care for women: A sub-study of the M@ NGO RCT. Women Birth. 2015;28:S14-S15.
38. Gidaszewski B, Khajehei M, Gibbs E, Chua SC. Comparison of the effect of caseload midwifery program and standard midwifery-led care on primiparous birth outcomes: A retrospective cohort matching study. Midwifery. 2019;69:10-16.
39. Giles W, Collins J, Ong F, MacDonald R. Antenatal care of low risk obstetric patients by midwives. A randomised controlled trial. Med J Aust. 1992;157:158-161.
40. Gottvall K, Waldenstrom U, Tingstig C, Grunewald C. In-hospital birth center with the same medical guidelines as standard care: a comparative study of obstetric interventions and outcomes. Birth. 2011;38:120-128.
41. Gutteridge K. Midwifery-led care for a low-risk cohort -- a clinical outcomes overview: over a three year period in a multicultural setting. MIDIRS Midwifery Digest. 2015;25:175-185.
42. Hailemeskel S, Alemu K, Christensson K, Tesfahun E, Lindgren H. Midwife-led continuity of care improved maternal and neonatal health outcomes in north Shoa zone, Amhara regional state, Ethiopia: A quasi-experimental study. Women Birth. 2021;03:03.
43. Hanley A, Davis D, Kurz E. Job satisfaction and sustainability of midwives working in caseload models of care: An integrative literature review. Women Birth. 2021;10:10.
44. Harvey S, Jarrell J, Brant R, Stainton C, Rach D. A randomized, controlled trial of nurse-midwifery care. Birth. 1996;23:128-135.
45. Harvey S, Rach D, Stainton MC, Jarrell J, Brant R. Evaluation of satisfaction with midwifery care. Midwifery. 2002;18:260-267.
46. Hicks C, Spurgeon P, Barwell F. Changing Childbirth: a pilot project. J Adv Nurs. 2003;42:617-628.
47. Hildingsson I. Women's Experiences of Care During Pregnancy in a Continuity of Midwifery Care Project in Rural Sweden. Int J Childbirth. 2021c;11:131-144.
48. Hildingsson I, Andersson E, Christensson K. Swedish women's expectations about antenatal care and change over time - a comparative study of two cohorts of women. Sex Reprod Healthc. 2014;5:51-57.
49. Hildingsson I, Karlstrom A, Larsson B. A continuity of care project with two on-call schedules: Findings from a rural area in Sweden. Sex Reprod Healthc. 2020a;26:100551.
50. Hildingsson I, Karlstrom A, Larsson B. Childbirth experience in women participating in a continuity of midwifery care project. Women Birth. 2021a;34:e255-e261.
51. Hildingsson I, Karlstrom A, Rubertsson C, Larsson B. Birth outcome in a caseload study conducted in a rural area of Sweden-a register based study. Sex Reprod Healthc. 2020b;24:100509.
52. Hildingsson I, Karlstrom A, Rubertsson C, Larsson B. Quality of intrapartum care assessed by women participating in a midwifery model of continuity of care. Eur J Midwifery. 2021b;5:11.
53. Hildingsson I, Rubertsson C, Karlstrom A, Haines H. A known midwife can make a difference for women with fear of childbirth- birth outcome and women's experiences of intrapartum care. Sex Reprod Healthc. 2019;21:33-38.
54. Holmes A, McGinley M, Turnbull D, Shields N, Hillan E. A consumer-driven quality assurance model for midwifery. Br J Midwifery. 1996;4:512-518.
55. Homer CS, Davis GK, Brodie PM. What do women feel about community-based antenatal care? Aust N Z J Public Health. 2000;24:590-595.
56. Homer CS, Matha DV, Jordan LG, Wills J, Davis GK. Community-based continuity of midwifery care versus standard hospital care: a cost analysis. Aust Health Rev. 2001;24:85-93.
57. Huber U, Sandall J. Continuity of carer, trust and breastfeeding. MIDIRS Midwifery Digest. 2006;16:445-449.
58. Hundley VA, Cruickshank FM, Lang GD, et al. Midwife managed delivery unit: a randomised controlled comparison with consultant led care. BMJ. 1994;309:1400-1404.
59. Hundley VA, Cruickshank FM, Milne JM, et al. Satisfaction and continuity of care: staff views of care in a midwife-managed delivery unit. Midwifery. 1995;11:163-173.
60. Hundley VA, Donaldson C, Lang GD, et al. Costs of intrapartum care in a midwife-managed delivery unit and a consultant-led labour ward. Midwifery. 1995;11:103-109.
61. Hundley VA, Milne JM, Glazener CM, Mollison J. Satisfaction and the three C's: continuity, choice and control. Women's views from a randomised controlled trial of midwife-led care. Br J Obstet Gynaecol. 1997;104:1273-1280.
62. Hunter B, Berg M, Lundgren I, Olafsdóttir OA, Kirkham M. Relationships: The hidden threads in the tapestry of maternity care. Midwifery. 2008;24:132-137.
63. Huynh M. Provider type and preterm birth in New York City births, 2009-2010. J Health Care Poor Underserved. 2014;25:1520-1529.
64. Iida M, Horiuchi S, Nagamori K. A comparison of midwife-led care versus obstetrician-led care for low-risk women in Japan. Women Birth. 2014;27:202-207.
65. Isaline G, Marie-Christine C, Rudy VT, Caroline D, Yvon E. An exploratory cost-effectiveness analysis: Comparison between a midwife-led birth unit and a standard obstetric unit within the same hospital in Belgium. Midwifery. 2019;75:117-126.
66. Jepsen I, Juul S, Foureur MJ, Sorensen EE, Nohr EA. Labour outcomes in caseload midwifery and standard care: a register-based cohort study. BMC Pregnancy Childbirth. 2018;18:481.
67. Jiang XM, Chen QY, Guo SB, et al. Effect of midwife-led care on birth outcomes of primiparas. Int J Nurs Pract. 2018;24:e12686.
68. Kataoka Y, Masuzawa Y, Kato C, Eto H. Maternal and neonatal outcomes in birth centers versus hospitals among women with low-risk pregnancies in Japan: A retrospective cohort study. Jpn J Nurs Sci. 2018;15:91-96.
69. Kenny C, Devane D, Normand C, Clarke M, Howard A, Begley C. A cost-comparison of midwife-led compared with consultant-led maternity care in Ireland (the MidU study). Midwifery. 2015;31:1032-1038.
70. Kenny P, Brodie P, Eckermann S, Hall J. Westmead Hospital team midwifery project evaluation. Final Report Sydney: Westmead Hospital. 1994.
71. Koto PS, Fahey J, Meier D, LeDrew M, Loring S. Relative effectiveness and cost-effectiveness of the midwifery-led care in Nova Scotia, Canada: A retrospective, cohort study. Midwifery. 2019;77:144-154.
72. Law YY, Lam KY. A randomized controlled trial comparing midwife-managed care and obstetrician-managed care for women assessed to be at low risk in the initial intrapartum period. J Obstet Gynaecol Res. 1999;25:107-112.
73. Lawton BA, Koch A, Stanley J, Geller SE. The effect of midwifery care on rates of cesarean delivery. Int J Gynaecol Obstet. 2013;123:213-216.
74. Lewis L, Hauck YL, Crichton C, Pemberton A, Spence M, Kelly G. An overview of the first 'no exit' midwifery group practice in a tertiary maternity hospital in Western Australia: Outcomes, satisfaction and perceptions of care. Women Birth. 2016;29:494-502.
75. Li Y, Townend J, Rowe R, Knight M, Brocklehurst P, Hollowell J. The effect of maternal age and planned place of birth on intrapartum outcomes in healthy women with straightforward pregnancies: secondary analysis of the Birthplace national prospective cohort study. BMJ Open. 2014;4:e004026.
76. McCormick M, Pollock W, Kapp S, Gerdtz M. Organizational strategies to optimize women's safety during labor and birth: A scoping review. Birth. 2021;48:285-300.
77. McGinley M, Turnbull D, Fyvie H, Johnstone I, MacLennan B. Midwifery development unit at Glasgow Royal Maternity Hospital. Br J Midwifery. 1995;3:362-371.
78. Mortensen B, Lieng M, Diep LM, Lukasse M, Atieh K, Fosse E. Improving Maternal and Neonatal Health by a Midwife-led Continuity Model of Care - An Observational Study in One Governmental Hospital in Palestine. EClinicalMedicine. 2019;10:84-91.
79. Mortensen B, Lukasse M, Diep LM, et al. Can a midwife-led continuity model improve maternal services in a low-resource setting? A non-randomised cluster intervention study in Palestine. BMJ Open. 2018;8:e019568.
80. O'Leary BD, Ciprike V. Are women attending a midwifery-led birthing center at increased risk of anal sphincter injury? Int Urogynecol J. 2020;31:583-589.
81. Offerhaus PM, de Jonge A, van der Pal-de Bruin KM, Hukkelhoven CW, Scheepers PL, Lagro-Janssen AL. Change in primary midwife-led care in the Netherlands in 2000-2008: a descriptive study of caesarean sections and other interventions among 789,795 low risk births. Midwifery. 2014;30:560-566.
82. Offerhaus PM, de Jonge A, van der Pal-de-Bruin KM, Hukkelhoven CW, Scheepers PL, Lagro-Janssen AL. Change in primary midwife-led care in the Netherlands in 2000-2008: A descriptive study of caesarean sections and other interventions among 807,437 low-risk births. Midwifery. 2015;31:648-654.
83. Pace CA, Crowther S, Lau A. Midwife experiences of providing continuity of carer: A qualitative systematic review. Women Birth. 2021;09:09.
84. Page L, Beake S, Vail A, McCourt C, Hewison J. Clinical outcomes of one-to-one midwifery practice. Br J Midwifery. 2001;9:700-706.
85. Permezel M, Milne KJ. Pregnancy outcome at term in low-risk population: study at a tertiary obstetric hospital. J Obstet Gynaecol Res. 2015;41:1171-1177.
86. Perriman N, Davis DL, Ferguson S. What women value in the midwifery continuity of care model: A systematic review with meta-synthesis. Midwifery. 2018;62:220-229.
87. Poskiene I, Vanagas G, Kirkilyte A, Nadisauskiene RJ. Comparison of vaginal birth outcomes in midwifery-led versus physician-led setting: A propensity score-matched analysis. Open Med. 2021;16:1537-1543.
88. Relph S, Delaney L, Melaugh A, et al. Costing the impact of interventions during pregnancy in the UK: a systematic review of economic evaluations. BMJ Open. 2020;10:e040022.
89. Ricchi A, Rossi F, Borgognoni P, et al. The midwifery-led care model: a continuity of care model in the birth path. Acta Biomed Ateneo Parmense. 2019;90:41-52.
90. Rowley MJ, Hensley MJ, Brinsmead MW, Wlodarczyk JH. Continuity of care by a midwife team versus routine care during pregnancy and birth: a randomised trial. Med J Aust. 1995;163:289-293.
91. Ryan P, Revill P, Devane D, Normand C. An assessment of the cost-effectiveness of midwife-led care in the United Kingdom. Midwifery. 2013;29:368-376.
92. Sandall J, Soltani H, Gates S, Shennan A, Devane D. Midwife-led continuity models versus other models of care for childbearing women. Cochrane Database Syst Rev. 2016;4:CD004667.
93. Seijmonsbergen-Schermers AE, Zondag DC, Nieuwenhuijze M, et al. Regional variations in childbirth interventions and their correlations with adverse outcomes, birthplace and care provider: A nationwide explorative study. PLoS One. 2020;15:e0229488.
94. Shields N, Holmes A, Cheyne H, et al. Knowing your midwife during labour. Br J Midwifery. 1999;7:504-510.
95. Shields N, Turnbull D, Reid M, Holmes A, McGinley M, Smith LN. Satisfaction with midwife-managed care in different time periods: a randomised controlled trial of 1299 women. Midwifery. 1998;14:85-93.
96. Spurgeon P, Hicks C, Barwell F. Antenatal, delivery and postnatal comparisons of maternal satisfaction with two pilot Changing Childbirth schemes compared with a traditional model of care. Midwifery. 2001;17:123-132.
97. Sutton F, McLauchlan M, Virtue C. Primary maternity care outcomes in New Zealand: a comparison of midwife and medical practitioner care. New Zealand College of Midwives Journal. 2002;26:5-8.
98. Suzuki S. Trend analysis of primary midwife-led delivery care at a Japanese perinatal center. Int J Med Sci. 2014;11:466-470.
99. Suzuki S. Recent Clinical Characteristics of Labors Using Three Japanese Systems of Midwife-Led Primary Delivery Care. Nurs Res Pract. 2016;2016:9101479.
100. Symon A, Winter C, Cochrane L. Exploration of preterm birth rates associated with different models of antenatal midwifery care in Scotland: Unmatched retrospective cohort analysis. Midwifery. 2015;31:590-596.
101. Talukdar S, Dingle K, Miller YD. A scoping review of evidence comparing models of maternity care in Australia. Midwifery. 2021;99:102973.
102. Thiessen K, Nickel N, Prior HJ, Banerjee A, Morris M, Robinson K. Maternity Outcomes in Manitoba Women: A Comparison between Midwifery-led Care and Physician-led Care at Birth. Birth. 2016;43:108-115.
103. Tracy SK, Welsh A, Hall B, et al. Caseload midwifery compared to standard or private obstetric care for first time mothers in a public teaching hospital in Australia: a cross sectional study of cost and birth outcomes. BMC Pregnancy Childbirth. 2014;14:46.
104. Tucker J. Team midwifery and continuity of care in hospital. Pract Midwife. 2000;3:24-30.
105. Turnbull D, Holmes A, Shields N, et al. Randomised, controlled trial of efficacy of midwife-managed care. Lancet. 1996;348:213-218.
106. Turnbull D, McGinley M, Fyvie H, et al. Implementation and evaluation of a midwifery development unit. Br J Midwifery. 1995;3:465-468.
107. Turnbull D, Shields N, McGinley M, et al. Professional issues. Can midwife-managed units improve continuity of care? Br J Midwifery. 1999;7:499‐503.
108. Waldenstrom U, Brown S, McLachlan H, Forster D, Brennecke S. Does team midwife care increase satisfaction with antenatal, intrapartum, and postpartum care? A randomized controlled trial. Birth. 2000;27:156-167.
109. Waldenstrom U, McLachlan H, Forster D, Brennecke S, Brown S. Team midwife care: maternal and infant outcomes. Aust N Z J Obstet Gynaecol. 2001;41:257-264.
110. Waldenström U, Nilsson CA. Women's satisfaction with birth center care: a randomized, controlled study. Birth. 1993;20:3-13.
111. Waldenström U, Nilsson CA. No effect of birth centre care on either duration or experience of breast feeding, but more complications: findings from a randomised controlled trial. Midwifery. 1994a;10:8-17.
112. Waldenstrom U, Nilsson CA. Experience of childbirth in birth center care. A randomized controlled study. Acta Obstet Gynecol Scand. 1994b;73:547-554.
113. Waldenström U, Nilsson CA. A randomized controlled study of birth center care versus standard maternity care: effects on women's health. Birth. 1997a;24:17-26.
114. Waldenstrom U, Nilsson CA, Winbladh B. The Stockholm birth centre trial: maternal and infant outcome. Br J Obstet Gynaecol. 1997b;104:410-418.
115. Wernham E, Gurney J, Stanley J, Ellison-Loschmann L, Sarfati D. A Comparison of Midwife-Led and Medical-Led Models of Care and Their Relationship to Adverse Fetal and Neonatal Outcomes: A Retrospective Cohort Study in New Zealand. PLoS Med. 2016;13:e1002134.
116. Wiegerinck MM, van der Goes BY, Ravelli AC, et al. Intrapartum and neonatal mortality in primary midwife-led and secondary obstetrician-led care in the Amsterdam region of the Netherlands: A retrospective cohort study. Midwifery. 2015;31:1168-1176.
117. Wiegerinck MMJ, Eskes M, van der Post JAM, Mol BW, Ravelli ACJ. Intrapartum and neonatal mortality in low-risk term women in midwife-led care and obstetrician-led care at the onset of labor: A national matched cohort study. Acta Obstet Gynecol Scand. 2020;99:546-554.
118. Wiegerinck MMJ, van der Goes BY, Ravelli ACJ, et al. Intrapartum and neonatal mortality among low-risk women in midwife-led versus obstetrician-led care in the Amsterdam region of the Netherlands: a propensity score matched study. BMJ Open. 2018;8:e018845.
119. Wilson KL, Sirois FM. Birth attendant choice and satisfaction with antenatal care: the role of birth philosophy, relational style, and health self‐efficacy. J Reprod Infant Psychol. 2010;28:69-83.
120. Young D, Lees A, Twaddle S. Professional issues. The costs to the NHS of maternity care: midwife-managed vs shared. Br J Midwifery. 1997b;5:465‐472.
121. Young D, Shields N, Holmes A, Turnbull D, Twaddle S. Aspects of antenatal care. A new style of midwife-managed antenatal care: costs and satisfaction. Br J Midwifery. 1997a;5:540‐545.
122. Zhang T, Liu C. Comparison between continuing midwifery care and standard maternity care in vaginal birth after cesarean. Pak J Med Sci. 2016;32:711-714.
123. Zhang Y, Xu K, Gong L, Sun Y, Ren F. The effect of continuous midwifery services on the delivery mode, labor progress, and nursing satisfaction of primiparas during natural deliveries. Am J Transl Res. 2021;13:7249-7255.
